# Rhizosphere Growth-Promoting Fungi of Healthy *Nicotiana tabacum* L.: A Systematic Approach to Boosting Plant Growth and Drought Resistance

**DOI:** 10.3390/microorganisms13030543

**Published:** 2025-02-27

**Authors:** Quanyu Yin, Zhao Feng, Zhichao Ren, Ao Li, Amit Jaisi, Mengquan Yang

**Affiliations:** 1 National Tobacco Cultivation, Physiology and Biochemistry Research Center, College of Tobacco Science, Henan Agricultural University, Zhengzhou 450046, China; 2 School of Pharmacy, Walailak University, Thasala, Nakhon Si Thammarat 80160, Thailand; 3 Biomass and Oil Palm Center of Excellence, Walailak University, Thasala, Nakhon Si Thammarat 80160, Thailand

**Keywords:** *Trichoderma harzianum*, drought stress, agronomic traits, physiological responses, biochemical indicators

## Abstract

Drought, exacerbated by global warming, poses a significant threat to crop growth and productivity. This study identified a strain of *Trichoderma harzianum* from the rhizosphere of healthy *Nicotiana tabacum* L. plants and evaluated its role in enhancing drought tolerance. The isolated strain effectively colonized plant roots and promoted the growth of *N. tabacum* L. To investigate its potential, *T. harzianum* was inoculated into plants under varying drought conditions, and its impact on growth, physiological responses, and drought resilience was assessed. Comprehensive analyses of agronomic traits, physiological parameters, enzyme activities, photosynthetic performance, osmoprotectant levels, and membrane lipid peroxidation revealed that *T. harzianum* inoculation (light drought with *T. harzianum*, moderate drought with *T. harzianum*, and severe drought with *T. harzianum* treatments) systematically improved plant development and drought resistance. These findings provide valuable insights and lay a foundation for developing innovative biofertilizers to enhance crop drought tolerance and sustainability.

## 1. Introduction

As the greenhouse effect intensifies, global temperatures continue to rise which is leading to an increased frequency of droughts [1]. This, in turn, causes significant reductions in crop yields, and in extreme cases, total crop failure. Water, being a crucial factor in plant growth and development, is indispensable. Its absence severely impacts the growth, yield, and quality of crops [2]. Consequently, studying plant responses to drought and developing new biofertilizers to enhance plant drought resistance have become prominent research topics [3,4].

In recent years, microbial inoculation has proven to be an effective method for promoting plant growth [5,6]. It can improve the soil’s microbial community structure, enhance the uptake of nutrients by plants, and increase their resistance to both biotic and abiotic stresses, thereby ensuring healthy growth [7,8,9,10]. Although the application of microbial inoculation in agriculture is widely recognized, its comprehensive effects on plants have not yet been fully explored. Recently, an increasing number of bacteria and fungi, such as *Bacillus* [11,12], *Pseudomonas* [13,14], arbuscular mycorrhizal fungi [12,15,16], and *Trichoderma* [17,18], have been shown to promote plant growth.

*Nicotiana tabacum* L., a widely cultivated economic crop, also serves as an important model organism in plant research [19]. Recently, *N. tabacum* L. has been developed as a chassis for synthetic biology, particularly for the production of natural products and antibodies [20,21,22]. Current research on *N. tabacum* L. primarily focuses on responses to biotic stressors (pathogens and insects) and abiotic stressors (UV radiation, drought, salinity, and waterlogging) [19,23,24]. However, studies on enhancing *N. tabacum* L.’s resilience to abiotic stress through microbial inoculation remain limited [25]. Therefore, it is crucial to investigate the changes in *N. tabacum* L. to elucidate the mechanisms by which *Trichoderma. harzianum* inoculation enhances drought resistance in this species.

This study isolated a strain of *T. harzianum* from the rhizosphere soil of healthy *N. tabacum* L. plants and evaluated its growth promoting effects on *N. tabacum* L. seedlings. Further experiments involving the inoculation of *N. tabacum* L. with *T. harzianum* demonstrated significant improvements in growth under drought stress conditions. Comprehensive analyses of plant phenotypes and physiological and biochemical indicators elucidated the mechanisms by which this plant growth-promoting fungus enhances drought resistance. This research developed a microbial inoculant capable of improving the drought tolerance of *N. tabacum* L., offering a potential solution to water scarcity issues exacerbated by global climate change.

## 2. Materials and Methods

### 2.1. Trichoderma Harzianum Isolation and Drought Treatment

Soil samples from the rhizosphere of healthy *N. tabacum* L. plants in a field affected by *Phytophthora nicotianae* (tobacco black shank disease) were collected to isolate *Trichoderma* strains. The strains were isolated from the soil samples using the dilution plating method and cultured on a PDA medium at 28 °C [26]. The growth promoting effects of the isolated *Trichoderma* strains on *N. tabacum* L. seedlings were evaluated. The colonization of the obtained *T. harzianum* in the plants was observed using acid fuchsin and fast green staining. The *T. harzianum* used in this study was isolated from the *N. tabacum* L. rhizosphere soil which was deposited at the China General Microbiological Culture Collection (CGMCC No. 23294).

**Figure 1:**
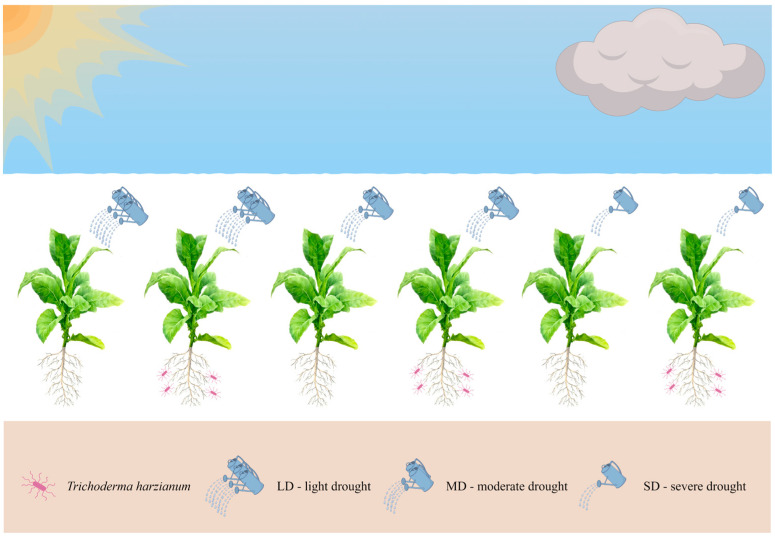
*Nicotiana tabacum* L. with six treatments. (LD, light drought; LDTh, light drought with *T. harzianum*; MD, moderate drought; MDTh, moderate drought with *T. harzianum*; SD, severe drought; SDTh, severe drought with *T. harzianum*).

The study was conducted using *Nicotiana tabacum* cultivar K362, and seeds provided by the College of Tobacco Science at Henan Agricultural University. Seedlings were germinated from seeds in controlled conditions and subsequently transplanted. To systematically study the mechanisms, a total of 180 well-grown plants at the same growth stage were selected for drought and *T. harzianum* treatments. Plants were divided into 6 groups, with 30 plants in each group, as follows: light drought (LD), light drought with *T. harzianum* (LDTh), moderate drought (MD), moderate drought with *T. harzianum* (MDTh), severe drought (SD), and severe drought with *T. harzianum* (SDTh) (Figure 1). Soil moisture levels were maintained as follows: CK (control): 70% of field capacity; LD (light drought): 60% of field capacity; MD (moderate drought): 50% of field capacity; SD (severe drought): 40% of field capacity. Soil moisture levels were monitored periodically to ensure consistency.

After 2 days of drought and *T. harzianum* root drenching treatment, the first assessment of plant growth was conducted, and samples were collected for further analyses. Sampling was performed every five days, with a total of four collections labeled as the 1st, 2nd, 3rd, and 4th. The collected samples were rapidly frozen in liquid nitrogen (N) and stored at −80 °C for subsequent physiological and biochemical analyses.

### 2.2. Agronomic Traits

Agronomic traits of the *N. tabacum* L. plants were measured using a tape measure, following the guidelines outlined in YC/T 142-2010, ’Methods for Survey and Measurement of Tobacco Agronomic Traits’ [27]. The parameters investigated included plant height, stem grith, maximum leaf length, maximum leaf width, and the number of effective leaves.

### 2.3. Root Development

The entire root system was carefully cleaned and scanned for appearance and morphology using an EPSON Expression 12000 XL plant root scanner (Epson, Nagano, Japan). The root system was then analyzed using WinRHIZO root analysis software (Regent Instruments, Quebec, QC, Canada, version Pro2007d) to determine the total root length, projection area, root surface area, root volume, root diameter, root tip number, branch number, and number of connections.

### 2.4. Relative Water Content of Leaves

A leaf sample (the third leaf from the top) was taken to measure the relative moisture content using the drying method [28]. Fresh *N. tabacum* L. leaves were first weighed, then saturated with water and weighed again. Finally, the leaves were dried and weighed. The relative water content was calculated as the percentage of the fresh leaf water content in relation to the saturated water content.

### 2.5. Root Activity

Root activity was measured using the TTC method [29]. During the measurement, 0.5 g of the root system was placed in a test tube with 10 mL of an equal mixture of 0.4% TTC solution and phosphate buffer. The mixture was incubated at 37 °C for 1 h in the dark, followed by the addition of 2 mL of 1 mol/L sulfuric acid to stop the reaction. The root was then removed, and surface moisture was absorbed. The root was placed in a test tube with 10 mL of methanol solution and kept in the dark at 37 °C for 2.5 h. A spectrophotometer (Spectronic 20 Genesys, Spectronic Instruments, Rochester, NY, USA) was used to measure the color at 485 nm, and the reduction amount of tetrazole was calculated.

### 2.6. Photosynthetic Parameters

The net photosynthetic rate, stomatal conductance, intercellular CO_2_ concentration, and transpiration rate were measured using a Li-6400 portable photosynthetic instrument (LI-COR Inc., Lincoln, NE, USA) between 9:00 a.m. and 11:30 a.m.

### 2.7. Photosynthetic Pigments

Following Wellburn’s method [30], the main veins were removed from the *N. tabacum* L. leaves, and 0.5 g of the leaves were weighed and placed in a 50 mL centrifuge tube. Then, 25 mL of 95% ethanol was added, and the tube was stored in a sealed, dark place for 24–36 h. A spectrophotometer (Spectronic 20 Genesys, Spectronic Instruments, Rochester, NY, USA) was used to measure the optical density (OD) at 665 nm, 649 nm, and 470 nm to determine the concentrations of chlorophyll a, chlorophyll b, and carotenoids, respectively. The concentrations were calculated using the formulas: C_a_ = 13.95D_665_ − 6.88D_649_; C_b_ = 24.96D_649_ − 7.32D_665_. The pigment content (mg/g) was then determined using the following formula: pigment content (mg/g) = (pigment concentration (mg/L) × extraction solution volume (mL) × dilution ratio)/sample mass (g).

### 2.8. Osmoprotectants

The same part of the leaf was frozen with liquid N and stored in a refrigerator at −80 °C. The contents of proline, soluble sugar, and soluble protein were determined using the Solarbio reagent kit, following the manufacturer’s protocol.

### 2.9. Leaf Protective Enzyme Activity

The same part of the leaf was frozen with liquid N and stored at −80 °C. The activities of catalase (CAT), peroxidase (POD), superoxide dismutase (SOD), and ascorbate peroxidase (APX) were measured using the Solarbio reagent kit.

### 2.10. Membrane Lipid Peroxidation Index

The same part of the leaf was frozen with liquid N and stored at −80 °C. The contents of malondialdehyde (MDA) and hydrogen peroxide (H_2_O_2_) were determined using the Solarbio reagent kit, following the manufacturer’s protocol.

### 2.11. Nitrate Reductase Activity

After being rapidly frozen in liquid N, the samples were stored at −80 °C. The nitrate reductase (NR) activity was measured using a UV–visible spectrophotometer, with the NR activity assay kit purchased from Beijing Solarbio Science & Technology Co., Ltd. (Beijing, China).

### 2.12. Data Processing

For the measurement of agronomic traits, 10 replications were performed, while physiological parameters were measured with 3 replications. The data were analyzed for statistical significance using one-way analysis of variance (ANOVA). A significance level of *p* < 0.05 was considered statistically significant.

The data were processed using Microsoft Excel 2019 (Microsoft Corporation, Redmond, WA, USA) and IBM SPSS Statistics 26.0 (IBM Corporation, Armonk, NY, USA) software for analysis of variance, and the significance of the data differences (lowercase letters on the bars in the figures) was tested. OriginPro 2021 (OriginLab Corporation, Northampton, MA, USA) was used for plotting.

## 3. Results

### 3.1. Plant Growth-Promoting Fungi Isolation and Its Effects Against Drought

**Figure 2:**
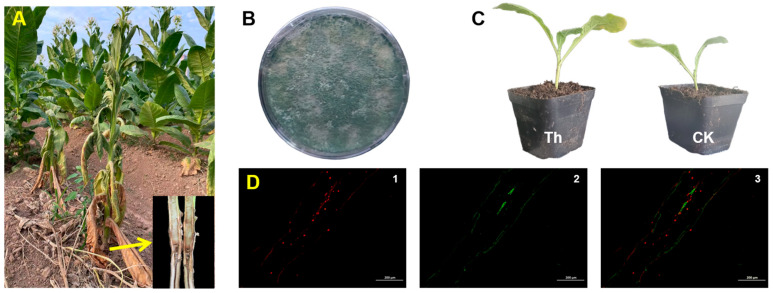
*Trichoderma harzianum* isolation from rhizosphere soil of healthy *N. tabacum* L. (**A**) Comparison of the healthy and infected *N. tabacum* L. in field; (**B**) *T. harzianum* cultured on potato dextrose agar media; (**C**) promoting-growth effect after *T. harzianum* inoculation (CK: control; Th: *T. harzianum*); (**D**) colonization of *T. harzianum* in *N. tabacum* L. roots.

Rhizosphere soil from healthy *N. tabacum* L. plants was collected from fields infected by tobacco black shank disease to isolate plant growth-promoting fungi (Figure 2A). A fungus capable of growing on the PDA medium was successfully isolated, identified as *T. harzianum* (Figure 2B), and deposited into the China General Microbiological Culture Collection (CGMCC No. 23294). Inoculation of *N. tabacum* L. seedlings with *T. harzianum* showed a significant growth-promoting effect under drought conditions (Figure 2C). Staining and microscopic observation of the inoculated *N. tabacum* L. roots revealed that *T. harzianum* (green) successfully colonized the roots (red), thereby enhancing plant growth and development (Figure 2D and Figure S4).

### 3.2. Agronomic Traits of N. tabacum L. After T. harzianum Inoculation During DS Treatment

**Figure 3:**
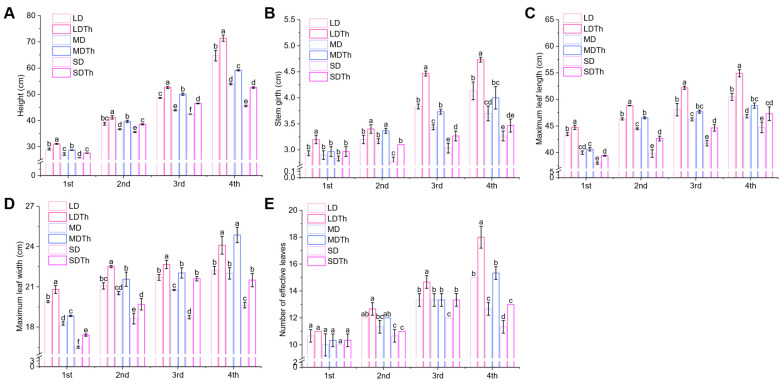
Enhancement of *N. tabacum* L.’s agronomic traits after *T. harzianum* inoculation under light drought (LD), moderate drought (MD), and severe drought (SD). (**A**) Height; (**B**) stem grith; (**C**) maximum leaf width; (**D**) maximum leaf length; (**E**) number of effective leaves.

Abiotic stress often significantly affects plant phenotypes. Therefore, agronomic traits were systematically recorded and analyzed across various treatments. A comparison of *T. harzianum* inoculation effects under varying drought conditions revealed that the inoculation significantly promoted plant growth and enhanced drought tolerance across light, moderate, and severe drought levels (Figure S1). Measurements revealed that under drought stress, *T. harzianum* inoculation had a notable impact on plant height (Figure 3A), stem grith (Figure 3B), maximum leaf length (Figure 3C), maximum leaf width (Figure 3D), and the number of effective leaves (Figure 3E). The metrics for *T. harzianum* treatments (LDTh, MDTh, SDTh) were consistently higher than those for the corresponding drought only groups (LD, MD, SD). Specifically, plant height increased by 10.30%, 9.64%, and 15.36%; stem girth by 16.52%, 8.74%, and 7.69%; maximum leaf length by 8.78%, 4.27%, and 5.74%; maximum leaf width by 8.40%, 13.03%, and 9.51%; and the number of effective leaves by 20.00%, 21.05%, and 14.71%, respectively (Figure 3).

### 3.3. Root System of N. tabacum L. After T. harzianum Inoculation During DS Treatment

**Figure 4:**
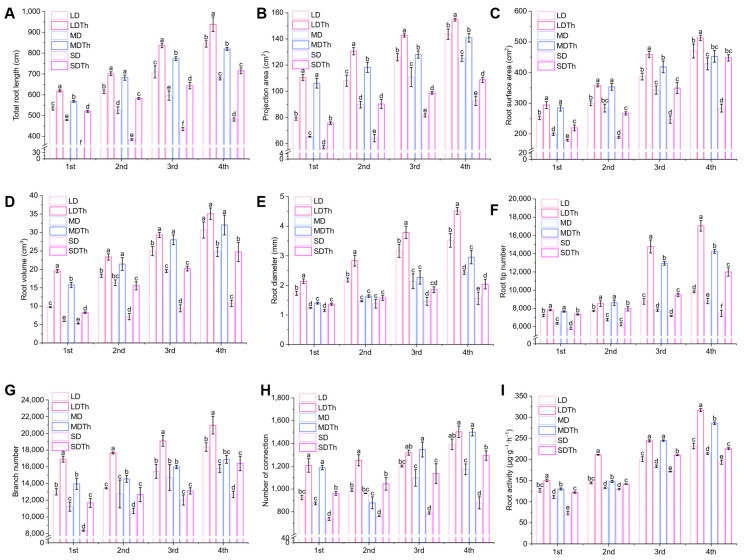
Enhancement of *N. tabacum* L.’s root growth and development after *T. harzianum* inoculation under light drought (LD), moderate drought (MD), and severe drought (SD). (**A**) Total root length; (**B**) root projection area; (**C**) root surface area; (**D**) root volume; (**E**) root diameter; (**F**) root tip member; (**G**) branch number; (**H**) number of connection; (**I**) root activity.

Roots, as essential organs for water and nutrient absorption, play a crucial role in providing plants with the necessary substances for growth. Observation of the root systems revealed that the roots of plants inoculated with *T. harzianum* were generally better developed than those of the non-inoculated plants (Figure S2). An assessment of root system parameters showed that *T. harzianum* inoculation under drought stress significantly affected total root length (Figure 4A), projection area (Figure 4B), root surface area (Figure 4C), root volume (Figure 4D), root diameter (Figure 4E), root tip number (Figure 4F), branch number (Figure 4G), root connectivity (Figure 4H), and root activity (Figure 4I). Compared to the corresponding drought-only treatments (LD, MD, and SD), all indicators in the *T. harzianum*-inoculated treatments (LDTh, MDTh, and SDTh) were consistently higher. Specifically, total root length increased by 17.90%, 29.87%, and 47.68%; root projection area by 39.86%, 62.97%, and 32.41%; root surface area by 19.68%, 24.40%, and 41.19%; root volume by 101.36%, 166.89%, and 55.22%; root diameter by 28.30%, 21.38%, and 30.46%; root tip number by 73.58%, 61.72%, and 60.67%; branch number by 30.07%, 24.32%, 39.66%; root connectivity by 30.43%, 36.10%, and 30.72%; root activity by 36.69%, 33.63%, 16.75%.

### 3.4. Photosynthetic Parameters of N. tabacum L. After T. harzianum Inoculation During DS Treatment

**Figure 5:**
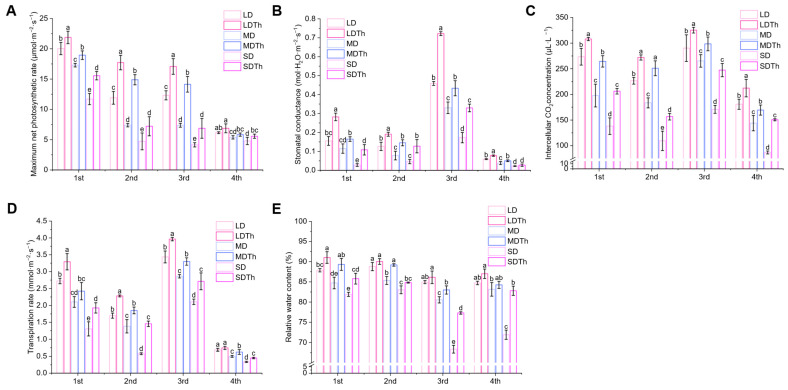
Effect of drought and *T. harzianum* inoculation on photosynthetic parameters. (**A**) Maximum net photosynthetic rate; (**B**) stomatal conductance; (**C**) intercellular CO_2_ concentration; (**D**) transpiration rate; (**E**) relative water content.

Photosynthesis is one of the most critical chemical reactions on Earth and is among the physiological processes in green plants most sensitive to drought. Measurements of photosynthetic parameters showed that inoculation with *T. harzianum* under drought stress significantly enhanced net photosynthetic rate (Figure 5A), stomatal conductance (Figure 5B), transpiration rate (Figure 5C), intercellular CO_2_ concentration (Figure 5D), and leaf relative water content (Figure 5E). In all cases, the indicators in the *Trichoderma*-treated groups (LDTh, MDTh, and SDTh) were higher than those in the corresponding drought-only groups (LD, MD, and SD). Specifically, the net photosynthetic rate increased by 48.89%, 101.56%, and 52.75%; stomatal conductance by 82.03%, 43.72%, and 288.78%; intercellular CO_2_ concentration by 20.01%, 36.79%, and 43.12%; transpiration rate by 34.29%, 33.93%, and 152.71%; and leaf relative water content by 3.60%, 5.43%, and 4.74%.

### 3.5. Photosynthetic Pigments of N. tabacum L. After T. harzianum Inoculation During DS Treatment

**Figure 6:**
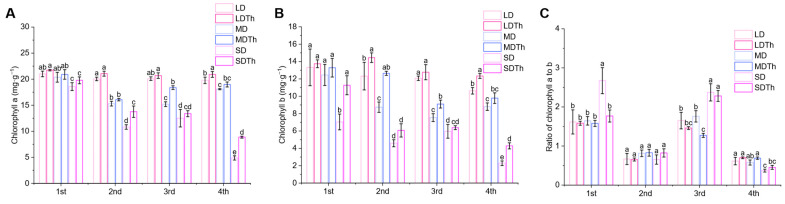
Effect of drought and *T. harzianum* inoculation on the photosynthetic pigments content of in *N. tabacum* L. leaves. (**A**) Chlorophyll *a*; (**B**) chlorophyll *b*; (**C**) ratio of chlorophyll *a* to *b*.

After measuring photosynthetic parameters, the associated pigments were also assessed. Inoculation with *T. harzianum* (LDTh, MDTh, and SDTh) resulted in higher levels of both chlorophyll *a* (Figure 6A) and chlorophyll *b* (Figure 6B) compared to the corresponding drought treatments (LD, MD, and SD). Chlorophyll *a* content increased by 5.79%, 5.00%, and 81.73%, while chlorophyll *b* content increased by 17.23%, 44.64%, and 32.30%. The chlorophyll a to b ratio ranged from 0.5 to 2.5 and was significantly higher under severe drought (SD) conditions during the first and third sampling periods (Figure 6C).

### 3.6. Osmoprotectants of N. tabacum L. After T. harzianum Inoculation During DS Treatment

**Figure 7:**
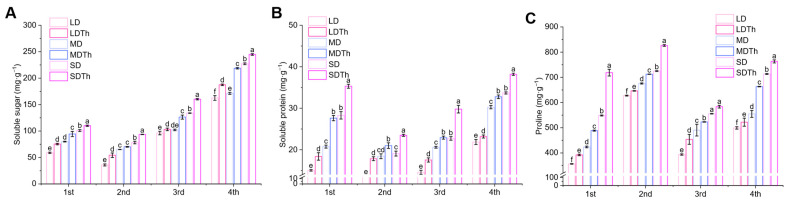
Effect of drought and *T. harzianum* inoculation on the content of osmoprotectants in *N. tabacum* L. leaves. (**A**) Soluble sugar; (**B**) soluble protein; (**C**) proline.

Plants often respond to abiotic stress by altering the levels of osmotic regulators. Therefore, we measured the contents of soluble sugar (Figure 7A), soluble protein (Figure 7B), and proline (Figure 7C). Inoculation with *T. harzianum* (LDTh, MDTh, and SDTh) resulted in higher levels of these substances compared to the corresponding drought treatments (LD, MD, and SD). Specifically, soluble sugar content increased by 51.38%, 7.80%, and 19.62%; soluble protein content increased by 22.39%, 33.11%, and 24.81%; and proline content increased by 10.01%, 15.28%, and 31.00%.

### 3.7. Membrane Lipid Peroxidation Index of N. tabacum L. After T. harzianum Inoculation During DS Treatment

**Figure 8:**
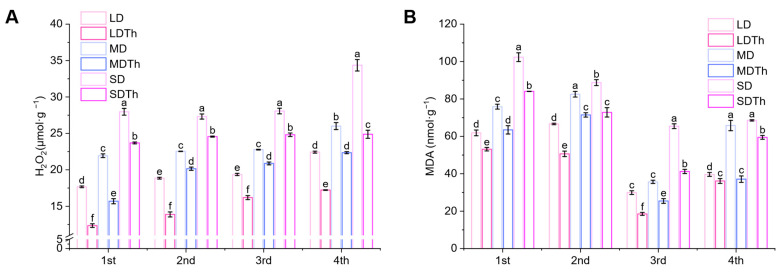
Effect of drought and *T. harzianum* inoculation on membrane lipid peroxidation index in *N. tabacum* L. leaves. (**A**) H_2_O_2_; (**B**) MDA.

The membrane lipid peroxidation index, mainly H_2_O_2_ (hydrogen peroxide) and MDA (malondialdehyde) contents, reflect the oxidative stress experienced by plants. Measurements revealed that H_2_O_2_ (Figure 8A) and MDA (Figure 8B) contents in *Trichoderma*-treated groups (LDTh, MDTh, and SDTh) were lower than those in the corresponding drought-treated groups (LD, MD, and SD). Specifically, H_2_O_2_ content decreased by 30.15%, 28.46%, and 15.29%, and MDA content decreased by 38.05%, 28.69%, and 37.00%.

### 3.8. Endogenous Protective Enzymes’ Activity of N. tabacum L. After T. harzianum Inoculation During DS Treatment

**Figure 9:**
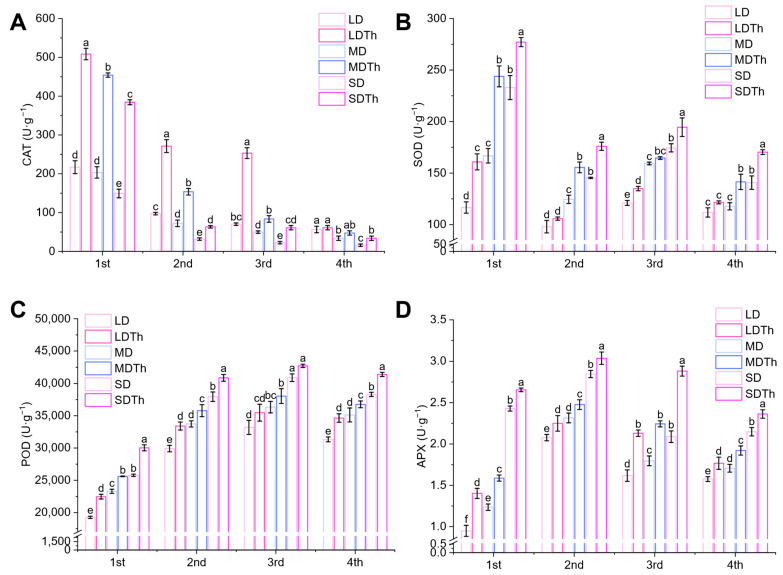
Effect of drought and *T. harzianum* inoculation on endogenous protective enzymes in *N. tabacum* L. leaves. (**A**) CAT; (**B**) SOD; (**C**) POD; (**D**) APX.

When plants face biotic and abiotic stresses, endogenous protective enzymes often become active. Therefore, we measured the activities of endogenous protective enzymes in *N. tabacum* L. under different treatments. The activities of CAT (catalase, Figure 9A), SOD (superoxide dismutase, Figure 9B), POD (peroxidase, Figure 9C), and APX (ascorbate peroxidase, Figure 9D) in the *T. harzianum*-treated groups (LDTh, MDTh, and SDTh) were higher than in the corresponding drought-treated groups (LD, MD, and SD). Specifically, CAT activity increased by 134.38%, 123.33%, and 157.58%; SOD activity increased by 37.98%, 46.22%, and 18.97%; POD activity increased by 16.67%, 9.90%, and 16.51%; and APX activity increased by 31.73%, 24.92%, and 38.00%.

### 3.9. Nitrate Reductase Activity of N. tabacum L. After T. harzianum Inoculation During DS Treatment

Nitrate reductase is a rate-limiting enzyme in the nitrogen assimilation of higher plants, directly regulating nitrate reduction and influencing both nitrogen metabolism and photosynthetic carbon metabolism. After measuring nitrate reductase activity, it was found that *T. harzianum* inoculation (LDTh, MDTh, and SDTh) resulted in higher nitrate reductase activity compared to the respective drought treatments (LD, MD, and SD), with increases of 51.24%, 91.84%, and 378.57%, respectively (Figure S3).

## 4. Discussion

*Trichoderma harzianum*, a fungus known for promoting plant growth, offers environmentally friendly advantages over chemical fertilizers and has been widely adopted as a biofertilizer [31]. In this study, a strain of *T. harzianum* was isolated, demonstrating both plant growth promotion and drought resistance. It was found to colonize plant roots effectively. Under drought conditions, *T. harzianum* inoculation was evaluated in detail to understand its mechanisms in promoting plant growth and enhancing drought resistance.

To date, many fungi, such as *Trichoderma* [17,18], arbuscular mycorrhizal fungi [12,15,16], and *Penicillium* [32,33,34], have been shown to promote plant growth. These fungi enhance seed germination, plant growth, root development, and photosynthesis, ultimately leading to increased crop yields. *T. harzianum*, as a natural endophytic biocontrol agent, needs to colonize plants to exert its defensive functions [31,35]. For instance, the colonization of *T. harzianum* in cannabis can promote its growth and development while increasing the content of the active compound cannabidiol (CBD) [36]. After inoculation with *Trichoderma* sp. T154 in grapes, the fungus can colonize the plant for the long term, located in the xylem, fibers, and parenchyma tissues, thereby providing resistance against *Phaeoacremonium minimum* infection [37]. Similarly, *Trichoderma* colonization in *Arabidopsis* roots enhances tolerance to abiotic stress and resistance to biotic stress [38]. Conversely, a reduction in *Trichoderma* colonization decreases its ability to enhance plant productivity. It has been shown that *Trichoderma* fungi improve resistance to both abiotic and biotic stresses, promote nutrient absorption, and stimulate plant growth by enhancing systemic resistance [31,35].

Drought, as one of the most critical environmental factors affecting plant growth, significantly impacts crop yield. This effect is exacerbated by the intensification of the greenhouse effect [39]. *Nicotiana tabacum* L., an important economic crop, plays a crucial role in the national economy [40]. Additionally, *N. tabacum* L. serves as a model plant for studying plant physiology and biochemistry, and as a chassis for synthetic biology [20,41,42]. Therefore, researching biocontrol agents that can promote *N. tabacum* L. growth and resist abiotic stress is urgently needed. This finding indicates that drought stress profoundly affects plant phenotypes, agronomic traits, and root development. However, inoculation with *Trichoderma* can mitigate drought-induced damage and promote *N. tabacum* L. growth.

Photosynthesis, the most important process in plant growth and development, is vital for crop yield formation [43]. Abiotic stresses, such as abnormal temperature, water scarcity, and nutrient deficiency, significantly limit photosynthesis, thereby threatening global food security [44,45]. For instance, potassium deficiency severely impacts photosynthesis, leading to reduced growth and crop yield [46]. High temperatures affect photosynthesis by influencing CO_2_ absorption, photochemical reactions, the turnover of D1 and D2 proteins, and chlorophyll biosynthesis [47]. The results demonstrate that under various levels of drought conditions, inoculating *N. tabacum* L. with *T. harzianum* significantly enhances photosynthesis-related parameters and positively impacts the accumulation of chlorophyll *a* and chlorophyll *b*.

When plants face extreme environmental conditions such as drought, high temperatures, or high salinity, their growth is severely inhibited, leading to significant oxidative stress and elevated membrane lipid peroxidation indicators [48]. To cope with these stresses, plants secrete osmotic regulatory substances to maintain osmotic balance and mitigate damage [49]. Additionally, plants have evolved a series of antioxidant enzyme systems, such as superoxide dismutase (SOD), catalase (CAT), peroxidase (POX), and ascorbate peroxidase (APX) [50]. Under varying degrees of drought stress, inoculation with *T. harzianum* significantly increased the activities of endogenous protective enzymes and the contents of osmotic regulatory substances (soluble sugars, soluble proteins, and proline) in *N. tabacum* L. Simultaneously, membrane lipid peroxidation indicators (e.g., H_2_O_2_ and MDA) showed a significant decrease. These results indicate that inoculation with *T. harzianum* can reduce the oxidative stress experienced by *N. tabacum* L. Furthermore, nitrate reductase, an important enzyme in nitrogen assimilation, plays a crucial role in plant growth and development [51]. Recent studies have shown that the activity of nitrate reductase is positively correlated with the concentration of auxin in plant roots, making it a key enzyme in regulating root structure [52]. Our findings demonstrate that inoculation with *T. harzianum* significantly enhances nitrate reductase activity, resulting in improved root development in *N. tabacum* L.

The broader application of *T. harzianum* as a biofertilizer holds significant promise for sustainable agriculture, especially in areas prone to water scarcity. As a biocontrol agent, *T. harzianum* not only helps in enhancing plant growth but also reduces the reliance on chemical fertilizers, which can have adverse environmental impacts. Its ability to enhance drought tolerance by stimulating the plant’s physiological responses can be a valuable tool in integrated crop management systems aimed at mitigating the effects of climate change. However, there are some limitations to this study that need to be addressed in future research. First, the study was conducted under controlled greenhouse conditions which do not fully replicate the complexity of field environments. Field trials are essential to confirm whether the observed benefits can be consistently achieved under real-world conditions, where factors such as soil type, microbial communities, and weather conditions can influence the outcomes. Furthermore, this study did not include a direct comparison with other biocontrol agents or microbial inoculants, which would provide a more comprehensive understanding of *T. harzianum*’s relative efficacy in enhancing drought resistance. Future studies should explore the synergy between *T. harzianum* and other beneficial microbes to determine optimal combinations for enhancing crop performance under stress conditions.

We conclude that the *T. harzianum* isolated in this study can systematically regulate the growth and development of *N. tabacum* L. under drought stress, enhancing its adaptability to drought. This study lays the foundation for the development of new, environmentally friendly, and green microbial biofertilizers for crop production.

## 5. Conclusions

From the rhizosphere of healthy *N. tabacum* L. plants in a field affected by black shank disease, the plant growth-promoting fungi *T. harzianum* was successfully isolated. Preliminary studies demonstrated its plant growth-promoting capabilities and its ability to colonize *N. tabacum* L. roots. Based on these findings, a comprehensive investigation was conducted into the effects of *T. harzianum* inoculation on plant phenotypes, agronomic traits, photosynthesis, osmotic regulatory substances, lipid peroxidation index, and enzyme activities. The research showed that under varying degrees of drought stress, *T. harzianum* inoculation significantly improved *N. tabacum* L. growth and drought resistance. By measuring various growth indicators, it was found that *T. harzianum* inoculation positively impacted plant growth and resistance to abiotic stress. This study helps researchers better understand plant responses to drought and the mechanisms by which *T. harzianum* promotes growth and enhances drought resistance, laying the foundation for developing eco-friendly biofertilizers.

## Data Availability

The original contributions presented in this study are included in the article/Supplementary Materials. Further inquiries can be directed to the corresponding author.

## Supplementary Materials

The following supporting information can be downloaded at: https://www.mdpi.com/article/10.3390/microorganisms13030543/s1, Figure S1: The plant growth status of *N. tabacum* L. under different drought conditions (w/o *T. harzianum* inoculation); Figure S2: The root system status of *N. tabacum* L. under different drought conditions (w/o *T. harzianum* inoculation); Figure S3: Nitrate reductase (NR) activity. Figure S4: Original pictures of Figure 2D.

## References

[1] Mora C., Spirandelli D., Franklin E.C., Lynham J., Kantar M.B., Miles W., Smith C.Z., Freel K., Moy J., Louis L.V. (2018). Broad threat to humanity from cumulative climate hazards intensified by greenhouse gas emissions. Nat. Clim. Change.

[2] Lesk C., Rowhani P., Ramankutty N. (2016). Influence of extreme weather disasters on global crop production. Nature.

[3] Chieb M., Gachomo E.W. (2023). The role of plant growth promoting rhizobacteria in plant drought stress responses. BMC Plant Biol..

[4] Anli M., Baslam M., Tahiri A., Raklami A., Symanczik S., Boutasknit A., Ait-El-Mokhtar M., Ben-Laouane R., Toubali S., Ait Rahou Y. (2020). Biofertilizers as Strategies to Improve Photosynthetic Apparatus, Growth, and Drought Stress Tolerance in the Date Palm. Front. Plant Sci..

[5] Li C., Chen X., Jia Z., Zhai L., Zhang B., Grüters U., Ma S., Qian J., Liu X., Zhang J. (2024). Meta-analysis reveals the effects of microbial inoculants on the biomass and diversity of soil microbial communities. Nat. Ecol. Evol..

[6] Cunha I.d.C.M.d., Silva A.V.R.d., Boleta E.H.M., Pellegrinetti T.A., Zagatto L.F.G., Zagatto S.d.S.S., Chaves M.G.d., Mendes R., Patreze C.M., Tsai S.M. (2024). The interplay between the inoculation of plant growth-promoting rhizobacteria and the rhizosphere microbiome and their impact on plant phenotype. Microbiol. Res..

[7] Li P., Dini-Andreote F., Jiang J. (2024). Exploiting microbial competition to promote plant health. Trends Plant Sci..

[8] Jiang Y., Zhang Y., Liu Y., Zhang J., Jiang M., Nong C., Chen J., Hou K., Chen Y., Wu W. (2024). Plant Growth-Promoting Rhizobacteria Are Key to Promoting the Growth and Furanocoumarin Synthesis of *Angelica dahurica* var. *formosana* under Low-Nitrogen Conditions. J. Agric. Food Chem..

[9] Wang J., Deng Z., Gao X., Long J., Wang Y., Wang W., Li C., He Y., Wu Z. (2024). Combined control of plant diseases by *Bacillus* subtilis SL44 and *Enterobacter hormaechei* Wu15. Sci. Total Environ..

[10] Ding Y., Gao X., Shu D., Siddique K.H.M., Song X., Wu P., Li C., Zhao X. (2024). Enhancing soil health and nutrient cycling through soil amendments: Improving the synergy of bacteria and fungi. Sci. Total Environ..

[11] Guo L., Zhang X., Zhao J., Zhang A., Pang Q. (2023). Enhancement of sulfur metabolism and antioxidant machinery confers *Bacillus* sp. Jrh14-10-induced alkaline stress tolerance in plant. Plant Physiol. Biochem..

[12] Khan W., Zhu Y., Khan A., Zhao L., Yang Y.-M., Wang N., Hao M., Ma Y., Nepal J., Ullah F. (2024). Above-and below-ground feedback loop of maize is jointly enhanced by plant growth-promoting rhizobacteria and arbuscular mycorrhizal fungi in drier soil. Sci. Total Environ..

[13] Papadopoulou A., Ainalidou A., Mellidou I., Karamanoli K. (2023). Metabolome and transcriptome reprogramming underlying tomato drought resistance triggered by a Pseudomonas strain. Plant Physiol. Biochem..

[14] Qiao Y., Wang Z., Sun H., Guo H., Song Y., Zhang H., Ruan Y., Xu Q., Huang Q., Shen Q. (2024). Synthetic community derived from grafted watermelon rhizosphere provides protection for ungrafted watermelon against Fusarium oxysporum via microbial synergistic effects. Microbiome.

[15] Adedayo A.A., Babalola O.O. (2023). Fungi That Promote Plant Growth in the Rhizosphere Boost Crop Growth. J. Fungi.

[16] Mohammadi E., Fattahi M., Barin M., Ashrafi-Saeidlou S. (2022). Arbuscular mycorrhiza and vermicompost alleviate drought stress and enhance yield, total flavonoid concentration, rutin content, and antioxidant activity of buckwheat (*Fagopyrum esculentum* Moench). S. Afr. J. Bot..

[17] Esparza-Reynoso S., Ruíz-Herrera L.F., Pelagio-Flores R., Macías-Rodríguez L.I., Martínez-Trujillo M., López-Coria M., Sánchez-Nieto S., Herrera-Estrella A., López-Bucio J. (2021). *Trichoderma* atroviride-emitted volatiles improve growth of Arabidopsis seedlings through modulation of sucrose transport and metabolism. Plant Cell Environ..

[18] Rebolledo-Prudencio O.G., Estrada-Rivera M., Dautt-Castro M., Arteaga-Vazquez M.A., Arenas-Huertero C., Rosendo-Vargas M.M., Jin H., Casas-Flores S. (2022). The small RNA-mediated gene silencing machinery is required in Arabidopsis for stimulation of growth, systemic disease resistance, and suppression of the nitrile-specifier gene NSP4 by *Trichoderma* atroviride. Plant J..

[19] Yin Q., Feng Z., Ren Z., Wang H., Wu D., Jaisi A., Yang M. (2024). Utilizing transcriptomics and metabolomics reveal drought tolerance mechanism in *Nicotiana tabacum*. bioRxiv.

[20] Molina-Hidalgo F.J., Vazquez-Vilar M., D’Andrea L., Demurtas O.C., Fraser P., Giuliano G., Bock R., Orzaez D., Goossens A. (2021). Engineering Metabolism in Nicotiana Species: A Promising Future. Trends Biotechnol..

[21] Mateos-Fernández R., Vacas S., Navarro-Fuertes I., Navarro-Llopis V., Orzáez D., Gianoglio S. (2023). Assessment of tobacco (*Nicotiana tabacum*) and *N. benthamiana* as biofactories of irregular monoterpenes for sustainable crop protection. Ind. Crops Prod..

[22] Zhao W., Zhou L.Y., Kong J., Huang Z.H., Gao Y.D., Zhang Z.X., Zhou Y.J., Wu R.Y., Xu H.J., An S.J. (2023). Expression of recombinant human Apolipoprotein A-I(Milano) in *Nicotiana tabacum*. Bioresour. Bioprocess..

[23] Saenz-de la O.D., Morales L.O., Strid A., Torres-Pacheco I., Guevara-Gonzalez R.G. (2021). Ultraviolet-B exposure and exogenous hydrogen peroxide application lead to cross-tolerance toward drought in *Nicotiana tabacum* L. Physiol. Plant.

[24] Wang L., Xu J.Y., Jia W., Chen Z., Xu Z.C. (2020). Chloride salinity in a chloride-sensitive plant: Focusing on photosynthesis, hormone synthesis and transduction in tobacco. Plant Physiol. Biochem..

[25] Shang X., Hui L., Jianlong Z., Hao Z., Cao C., Le H., Weimin Z., Yang L., Gao Y., Hou X. (2023). The application of plant growth-promoting rhizobacteria enhances the tolerance of tobacco seedling to salt stress. Ecotoxicol. Environ. Saf..

[26] Wang X., Wang C., Li Q., Zhang J., Ji C., Sui J., Liu Z., Song X., Liu X. (2018). Isolation and characterization of antagonistic bacteria with the potential for biocontrol of soil-borne wheat diseases. J. Appl. Microbiol..

[27] Dai J., Wen D., Li H., Yang J., Rao X., Yang Y., Yang J., Yang C., Yu J. (2024). Effect of hydrogen sulfide (H(2)S) on the growth and development of tobacco seedlings in absence of stress. BMC Plant Biol..

[28] Weatherley P.E. (1950). A Convenient Volumenometer for Biological Work. J. Exp. Bot..

[29] Wang H., He Y., Zheng Q., Yang Q., Wang J., Zhu J., Zhan X. (2024). Toxicity of photoaged polyvinyl chloride microplastics to wheat seedling roots. J. Hazard. Mater..

[30] Wellburn A.R. (1994). The Spectral Determination of Chlorophylls a and b, as well as Total Carotenoids, Using Various Solvents with Spectrophotometers of Different Resolution. J. Plant Physiol..

[31] Woo S.L., Hermosa R., Lorito M., Monte E. (2023). *Trichoderma*: A multipurpose, plant-beneficial microorganism for eco-sustainable agriculture. Nat. Rev. Microbiol..

[32] Kaur R., Saxena S. (2023). Penicillium citrinum, a Drought-Tolerant Endophytic Fungus Isolated from Wheat (*Triticum aestivum* L.) Leaves with Plant Growth-Promoting Abilities. Curr. Microbiol..

[33] Tarroum M., Romdhane W.B., Al-Qurainy F., Ali A.A.M., Al-Doss A., Fki L., Hassairi A. (2022). A novel PGPF *Penicillium olsonii* isolated from the rhizosphere of Aeluropus littoralis promotes plant growth, enhances salt stress tolerance, and reduces chemical fertilizers inputs in hydroponic system. Front. Microbiol..

[34] Ai M., Han F., Yang X., Chu H., Luo C., Tan S., Lv S., Qin M., Xie G. (2023). Endophytic *Penicillium oxalicum* CX-1 prevented *Phytophthora cactorum* blight on *Salvia miltiorrhiza* and promoted plant growth. J. Appl. Microbiol..

[35] Poveda J., Eugui D., Abril-Urias P., Sharma A.K., Sharma P. (2020). Could *Trichoderma* Be a Plant Pathogen? Successful Root Colonization. Trichoderma: Host Pathogen Interactions and Applications.

[36] Kakabouki I., Tataridas A., Mavroeidis A., Kousta A., Karydogianni S., Zisi C., Kouneli V., Konstantinou A., Folina A., Konstantas A. (2021). Effect of Colonization of *Trichoderma harzianum* on Growth Development and CBD Content of Hemp (*Cannabis sativa* L.). Microorganisms.

[37] Carro-Huerga G., Compant S., Gorfer M., Cardoza R.E., Schmoll M., Gutierrez S., Casquero P.A. (2020). Colonization of *Vitis vinifera* L. by the Endophyte *Trichoderma* sp. Strain T154: Biocontrol Activity Against *Phaeoacremonium minimum*. Front. Plant Sci..

[38] Poveda J. (2021). Glucosinolates profile of Arabidopsis thaliana modified root colonization of *Trichoderma* species. Biol. Control.

[39] Vadez V., Grondin A., Chenu K., Henry A., Laplaze L., Millet E.J., Carminati A. (2024). Crop traits and production under drought. Nat. Rev. Earth Environ..

[40] Gui Z.-Q., Yuan X.-L., Yang J., Du Y.-M., Zhang P. (2024). An updated review on chemical constituents from *Nicotiana tabacum* L.: Chemical diversity and pharmacological properties. Ind. Crops Prod..

[41] Gossart N., Berhin A., Sergeant K., Alam I., Andre C., Hausman J.F., Boutry M., Hachez C. (2023). Engineering *Nicotiana tabacum* trichomes for triterpenic acid production. Plant Sci..

[42] Liu X., Zhang P., Zhao Q., Huang A.C. (2023). Making small molecules in plants: A chassis for synthetic biology-based production of plant natural products. J. Integr. Plant Biol..

[43] Chauhan J., Prathibha M.D., Singh P., Choyal P., Mishra U.N., Saha D., Kumar R., Anuragi H., Pandey S., Bose B. (2023). Plant photosynthesis under abiotic stresses: Damages, adaptive, and signaling mechanisms. Plant Stress..

[44] Hu W., Tian S.B., Di Q., Duan S.H., Dai K. (2018). Effects of exogenous calcium on mesophyll cell ultrastructure, gas exchange, and photosystem II in tobacco (*Nicotiana tabacum* Linn.) under drought stress. Photosynthetica.

[45] Li Y., Jiang F., Niu L., Wang G., Yin J., Song X., Ottosen C.O., Rosenqvist E., Mittler R., Wu Z. (2024). Synergistic regulation at physiological, transcriptional and metabolic levels in tomato plants subjected to a combination of salt and heat stress. Plant J..

[46] Imtiaz H., Mir A.R., Corpas F.J., Hayat S. (2023). Impact of potassium starvation on the uptake, transportation, photosynthesis, and abiotic stress tolerance. Plant Growth Regul..

[47] Zahra N., Hafeez M.B., Ghaffar A., Kausar A., Zeidi M.A., Siddique K.H.M., Farooq M. (2023). Plant photosynthesis under heat stress: Effects and management. Environ. Exp. Bot..

[48] Zulfiqar F., Akram N.A., Ashraf M. (2019). Osmoprotection in plants under abiotic stresses: New insights into a classical phenomenon. Planta.

[49] Ahmad F., Singh A., Kamal A. (2020). Osmoprotective Role of Sugar in Mitigating Abiotic Stress in Plants. Protective Chemical Agents in the Amelioration of Plant Abiotic Stress.

[50] Rajput V.D., Harish, Singh R.K., Verma K.K., Sharma L., Quiroz-Figueroa F.R., Meena M., Gour V.S., Minkina T., Sushkova S. (2021). Recent Developments in Enzymatic Antioxidant Defence Mechanism in Plants with Special Reference to Abiotic Stress. Biology.

[51] Liu X., Hu B., Chu C. (2022). Nitrogen assimilation in plants: Current status and future prospects. J. Genet. Genom..

[52] Fu Y.-F., Zhang Z.-W., Yang X.-Y., Wang C.-Q., Lan T., Tang X.-Y., Chen G.-D., Zeng J., Yuan S. (2020). Nitrate reductase is a key enzyme responsible for nitrogen-regulated auxin accumulation in Arabidopsis roots. Biochem. Biophys. Res. Commun..

