# Supplementary material for: Rhizosphere Growth-Promoting Fungi of Healthy Nicotiana tabacum L.: A Systematic Approach to Boosting Plant Growth and Drought Resistance
Source: Microorganisms. 2025 Feb 27;13(3):543. doi: 10.3390/microorganisms13030543 (PMC11944967; doi:10.3390/microorganisms13030543)
Supplement: Supplementary file 1 [file microorganisms-13-00543-s001.zip › microorganisms-3430318-supplementary.pdf]

Supplementary Materials

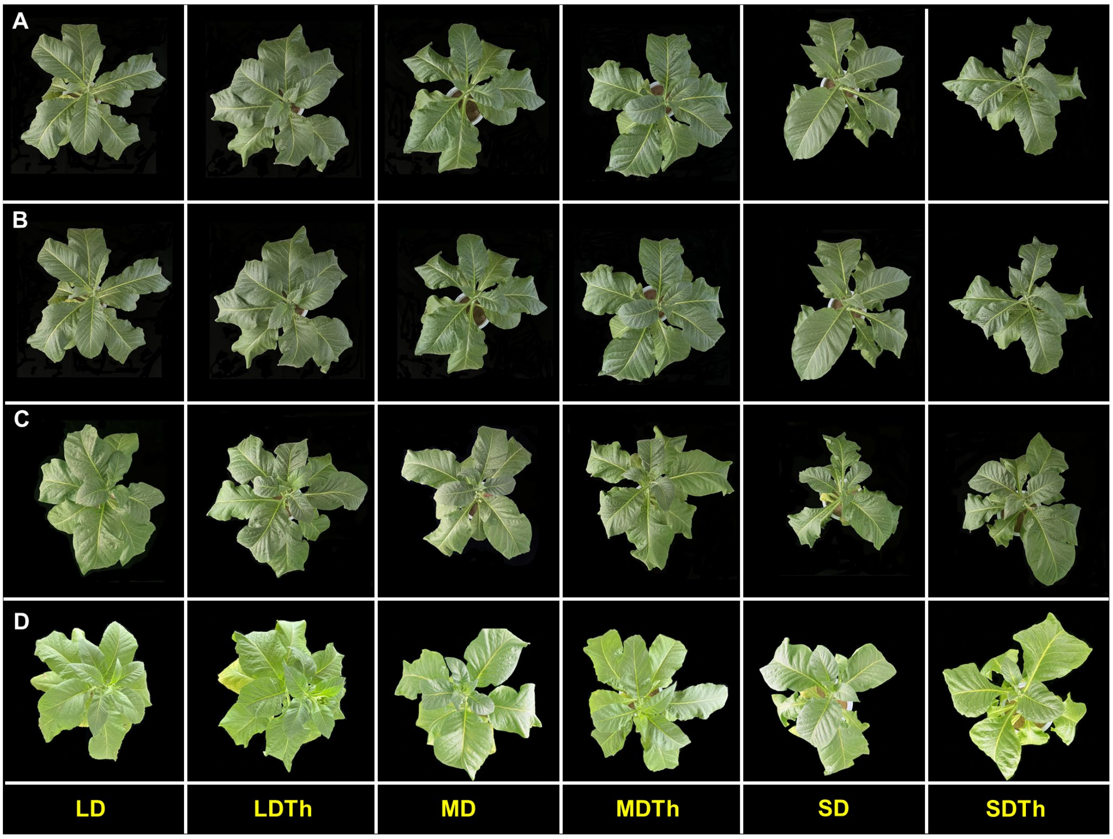

Figure S1. The plant growth status of *N. tabacum* under different drought conditions (w/o *T. harzianum* inoculation). A. the first time sampling (1st); B. the second time sampling (2nd); C. the third time sampling (3rd); D. the fourth time sampling (4th). Drought conditions: well-watered (CK); light drought (LD); moderate drought (MD) and severe drought (SD).

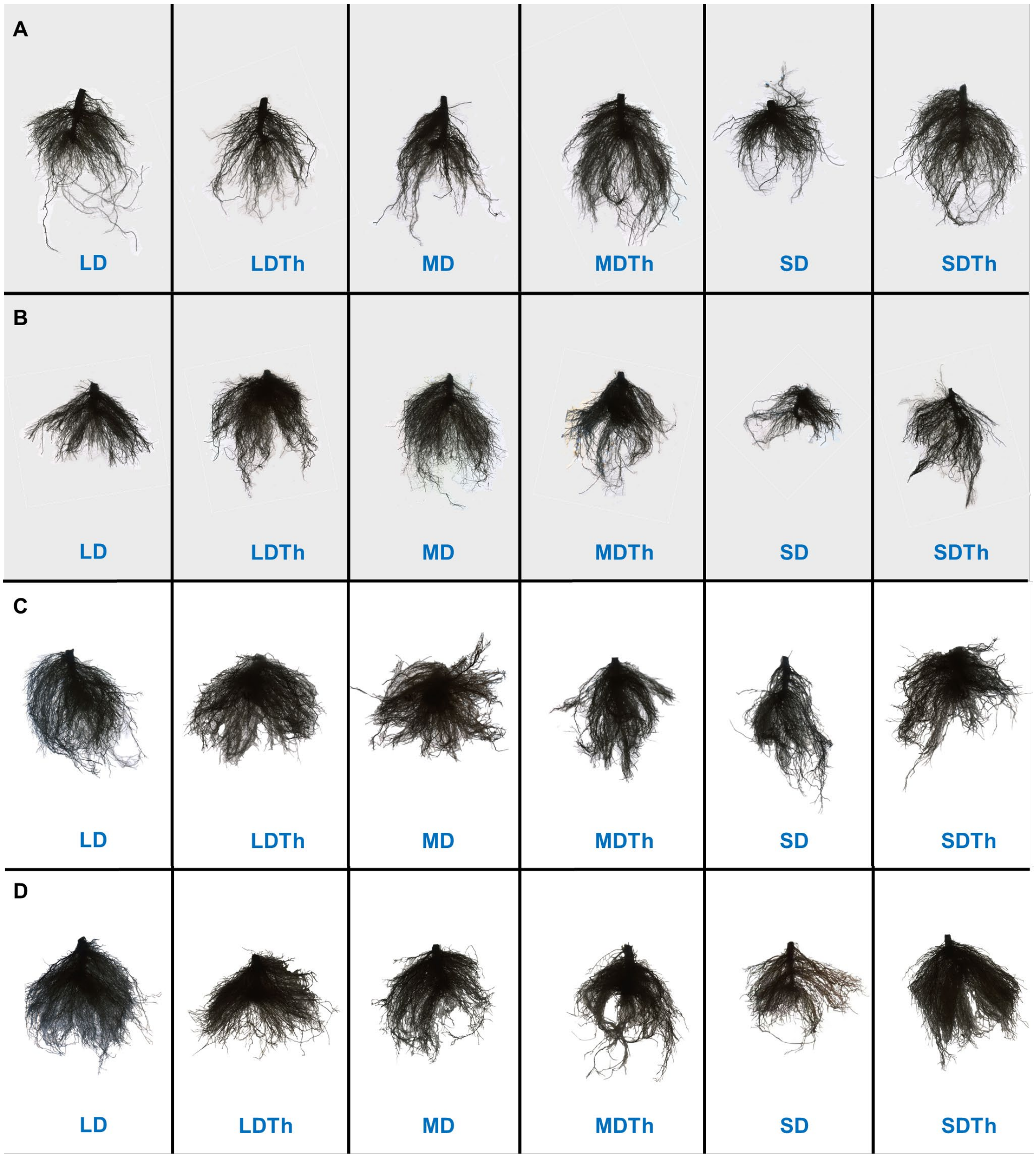

Figure S2. The root system status of *N. tabacum* under different drought conditions (w/o *T. harzianum* inoculation). A. the first time sampling (1st); B. the second time sampling (2nd); C. the third time sampling (3rd); D. the fourth time sampling (4th). Drought conditions: well-watered (CK); light drought (LD); moderate drought (MD) and severe drought (SD).

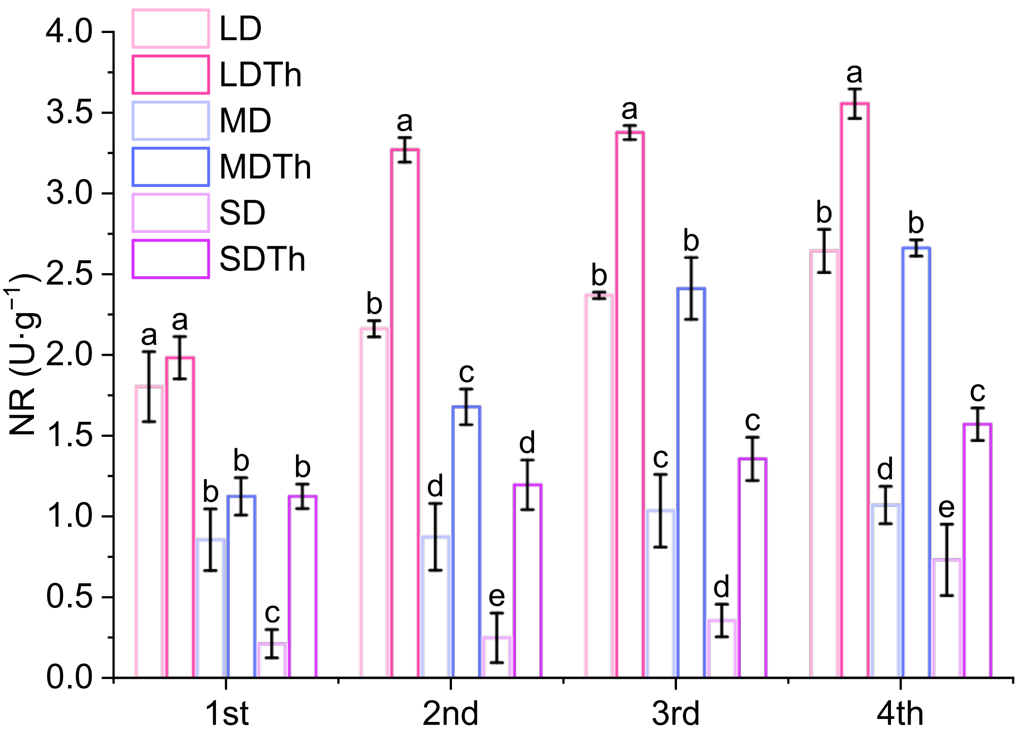

Figure S3. Nitrate reductase (NR) activity.

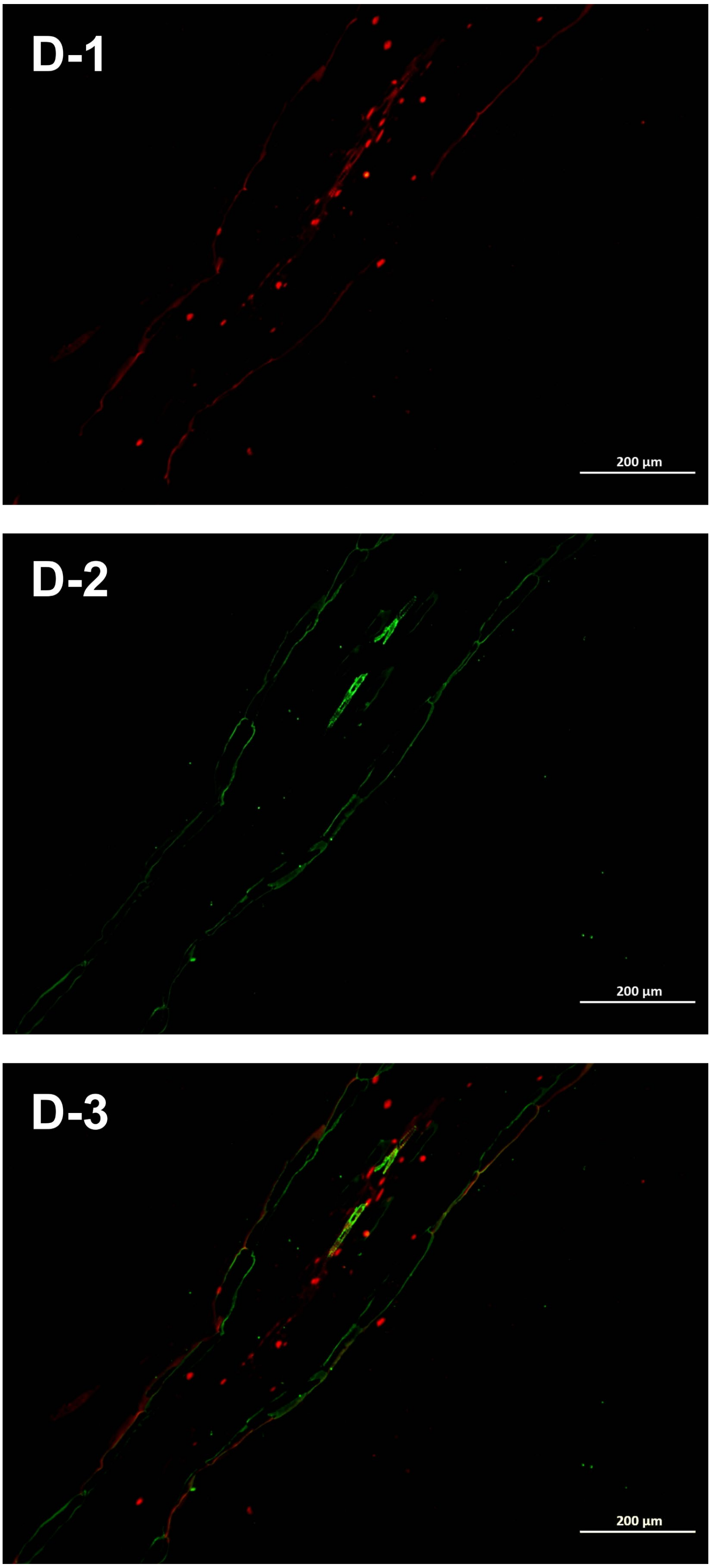

Figure S4. Original pictures of Figure 2D.
